# Prevalence of the pathogenic chytrid fungus, *Batrachochytrium dendrobatidis*, in an endangered population of northern leopard frogs, *Rana pipiens*

**DOI:** 10.1186/1472-6785-10-6

**Published:** 2010-03-04

**Authors:** Maarten J Voordouw, Doug Adama, Barb Houston, Purnima Govindarajulu, John Robinson

**Affiliations:** 1 Department of Biology, University of Victoria, PO Box 3020, Station CSC, Victoria, British Columbia, V8W 3N5, Canada; 2 BC Hydro, Unit 1 1007 11th Avenue, Golden, British Columbia, V0A 1H0, Canada; 3 Fish and Wildlife Compensation Program - Columbia Basin, 103-333 Victoria Street, Nelson, British Columbia, V1L 4K3, Canada; 4 BC Ministry of Environment, PO Box 9338 Stn Prov Govt, Victoria, British Columbia, V8W 9M1, Canada; 5 Animal Health Centre, BC Ministry of Agriculture & Lands, 1767 Angus Campbell Road, Abbotsford, British Columbia, V3G 2M3, Canada

## Abstract

**Background:**

Emerging infectious diseases threaten naïve host populations with extinction. Chytridiomycosis, an emerging infectious disease of amphibians, is caused by the pathogenic fungus *Batrachochytrium dendrobatidis *(*Bd*) and has been linked to global declines in amphibians.

**Results:**

We monitored the prevalence of *Bd *for four years in the Northern leopard frog, *Rana pipiens*, which is critically imperiled in British Columbia (BC), Canada. The prevalence of *Bd *initially increased and then remained constant over the last three years of the study. Young of the year emerging from breeding ponds in summer were rarely infected with *Bd*. Some individuals cleared their *Bd *infections and the return rate between infected and uninfected individuals was not significantly different.

**Conclusions:**

The BC population of *R. pipiens *appears to have evolved a level of resistance that allows it to co-exist with *Bd*. However, this small population of *R. pipiens *remains vulnerable to extinction.

## Background

Infectious diseases can have devastating consequences for immunologically naïve host populations. Human infectious diseases such as measles, tuberculosis, and smallpox are believed to have killed as many as 95% of all Native Americans in the two centuries following the first contact with Europeans [1]. In the UK, the decline of the native red squirrel and its replacement by the introduced grey squirrel is believed to be mediated by the parapoxvirus [2]. Small, inbred host populations are especially vulnerable to new pathogens due to a lack of genetic variation [3]. For example, the introduction of avian malaria has decimated populations of Hawaiian birds [4]. The Christmas Island rat, *Rattus macleari*, went extinct in less than five years following contact with the black rat, *Rattus rattus*, which carried a pathogenic trematode [5]. In this study, we investigated the role of chytridiomycosis, a recently emerged infectious disease of amphibians, in the decline of a small, endangered population of the northern leopard frog, *Rana pipiens*.

Chytridiomycosis is a skin disease of amphibians that is caused by the chytridiomycete fungus, *Batrachochytrium dendrobatidis *[6,7]. The waterborne zoospores of this fungus attack keratinized tissues including the skin of post-metamorphic individuals and the mouthparts of tadpoles. In post-metamorphic individuals, chytridiomycosis causes hyperkeratosis (a marked thickening of the *stratum corneum*) and excessive skin sloughing, which can impair cutaneous respiration and osmoregulation and result in death [6]. Chytrid zoospores have limited swimming ability (~2 cm) [8] and the fungus appears to depend on water flow or host movement for long distance dispersal [9]. The fungus grows best between 17 and 25°C and cannot grow at air temperatures higher than 28°C [7]. Recent work suggests that Bd may produce tiny, non-pathogenic resting spores that attach to the amphibian skin surface but without causing disease [10].

Chytridiomycosis is believed to be responsible for the mass mortality and extinction events of amphibian populations in Australia [11], Panama and Costa Rica [12,13]. Most of the chytridiomycosis-related die-offs have occurred in amphibians that breed in permanent water bodies reflecting the aquatic nature of the disease [12]. In Queensland, Australia, amphibians that breed in ephemeral water bodies or terrestrial environments were seldom infected with *Bd *[14]. Similarly, a survey in Maine, USA, found that infection prevalence in species that hibernate in terrestrial habitats was almost three times lower than that in species that hibernate in aquatic habitats [15]. The disease is generally less virulent in tadpoles than post-metamorphic individuals. Mass mortality events in Arizona and California have found apparently healthy larvae in the presence of dead or dying adult frogs [16,17]. There is variation among and within species in susceptibility to the disease [18-21].

The northern leopard frog, *Rana pipiens*, is a medium-sized, semi-terrestrial frog that is widely distributed in North America [22,23]. *R. pipiens *emerges from its overwintering habitat in early spring and adults move to the breeding ponds. Mating and egg laying occurs from mid-April to early June. Tadpoles transform into post-metamorphic froglets in late July and disperse away from their natal pond over the next several weeks. After breeding, adults venture into adjacent upland areas to forage. In late August to September, adults, juveniles and young of the year head to their overwintering habitat. We therefore expect *R. pipiens *to be most vulnerable to *Bd *during breeding (April to June) and overwintering (October to March) due to their aquatic nature at these times.

The Committee on the Status of Endangered Wildlife in Canada (COSEWIC) has listed *R. pipiens *as Endangered in British Columbia because only two small populations remain. Both populations are in the southeastern part of the province, one naturally occurring and one recently reintroduced as part of a recovery effort [24]. Surveyors estimated that between 2000 to 2005 the BC population declined by 50% and found that *R. pipiens *was infected with *Bd *[24,25]. In 2003, we began a mark-recapture study of *R. pipiens*. The purpose of this study was to monitor the prevalence of *Bd *over time and to determine whether season and stage class influenced infection levels. The mark-recapture design of the study allowed us to test whether *R. pipiens *can clear their *Bd *infection as demonstrated in previous studies in Australia [26,27]. In this study we also compared the sensitivity of three different tissue-sampling methods in determining whether a frog was infected with *Bd*.

## Methods

### Study area

The study area included the Creston Valley and Bummers Flats Wildlife Management Areas (CVWMA and BFWMA) in southeast British Columbia, Canada and is described in detail by Adama and Beaucher [24]. The CVWMA occupies 6,885 ha and the BFWMA occupies 850 ha.

### Survey Methods

The survey started in the fall of 2003. From 2004 to 2007 surveyors visited the CVWMA and the BFWMA in the spring, summer and fall. The annual sampling effort in 2003, 2004, 2005, 2006, and 2007 (for the CVWMA and the BFWMA combined) was 119, 160, 200, 159, and 49 visits, respectively, which took 199, 308, 417, 314, and 108 person hours. Surveyors encountered and captured 320 *R. pipiens *during nocturnal calling surveys, egg mass surveys and visual encounter surveys [for details see [24]] and took 401 tissue samples.

For each *R. pipiens *capture, surveyors took a photo of the dorsal side, which has a unique pattern of large, dark circular spots. These photos were used to identify individuals and to determine recaptures within and among subsequent years. Other studies have used spot patterns to successfully identify individual leopard frogs [28].

For each captured animal, surveyors recorded its GPS coordinates and measured its snout-vent length and body weight. We used body weight and season of capture to assign individuals to one of three stage classes: young of the year, juvenile, and adult. The three stage classes were categorized as follows: young of the year weighed less than 35 grams in the summer and fall. A juvenile weighed between 35 and 50 grams in the summer and fall or weighed less than 50 grams in the spring. An adult was any frog that weighed more than 50 grams. Captured animals were also checked for symptoms of chytridiomycosis, which include sloughing skin, redness, lethargy, abnormal body positioning, loss of righting reflex, and vascularization.

### Tissue sampling and PCR test for *Bd*

To determine whether the animal was infected with *Bd*, we collected tissue samples for PCR analysis. Tissue samples were collected using three different methods including toe clips, bag rinses, and swabs. In the toe clip method, we cut the terminal phalange of the fourth toe of the animal's right hind foot. In the bag rinse method, an animal was lightly "massaged" within a (single use) zip lock bag to collect tissue and the bag was subsequently rinsed out with ethanol. In the swab method, we used a sterile cotton tip swab (#018-460 AMG Medical Inc) to swab the abdomen, thighs, groin and feet of the animal 10 to 20 times. The tissue sampling method changed over the course of the study. In 2003 we used toe clips because *Bd *was primarily diagnosed using histological techniques [6]. In 2004, we switched to less invasive swab and bag rinse methods because PCR became the standard method of identifying *Bd *[29] and because *R. pipiens *is an endangered species. From 2003 to 2006 all swabs were preserved in ethanol. In 2007, swabs were stored in tubes without ethanol (i.e., dry swabs). We changed the swab storage protocol because extracting DNA from dry swabs was less time consuming than extracting DNA from swabs stored in ethanol. Hyatt *et al *[30] demonstrated that dry swabs and swabs stored in ethanol are equally effective at detecting *Bd *DNA. Tissue samples were sent to the Animal Health Centre of the Ministry of Agriculture in Abbotsford, BC where they were tested for *Bd *using PCR. We followed the methods of Boyle *et al*. [29] except that we did not construct a standard curve. We therefore cannot determine zoospore load and the PCR data consisted of whether a frog was infected or not. Unfortunately, the tissue samples from 2004 were lost and so we have PCR data for 2003, 2005, 2006 and 2007.

### Statistical Methods

#### Independence of data and pseudo-replication

There are three levels of replication in the survey: (1) animal, (2) capture occasion, and (3) tissue sample. An animal refers to a unique *R. pipiens *individual. A capture occasion refers to the date that an animal was captured (i.e. the same animal can be captured on multiple occasions). A tissue sample refers to the fact that sometimes we obtained multiple tissue samples from the same capture occasion using different methods (i.e. bag rinse, swab, toe clip). The recapture rate of *R. pipiens *was low (31 recaptures/320 captures) and we therefore treated all 320 captures as independent. For capture occasions with multiple tissue samples, the animal was considered *Bd*-positive if at least one of the tissue samples tested positive for *Bd*.

#### Sensitivity of 3 tissue-sampling methods

The subset of capture occasions with multiple tissue samples allowed us to compare the sensitivity of the three tissue sampling methods. We used the Chi-square test to determine statistical significance.

#### Prevalence of *Batrachochytrium dendrobatidis *in *Rana pipiens*

The prevalence of *Bd *(i.e. the proportion of infected animals) is binomial data because an animal is either infected or not. We used generalized linear models (GLM) with a binomial error distribution to model the prevalence of *Bd *in *R. pipiens *as a function of the following five factors: season, year, stage class, location and tissue-sampling method. The levels of each factor are as follows: season (spring = April to June, summer = July to August, fall = September to October), year (2003, 2005, 2006, 2007), stage class (young of year, juveniles, adults), location (CVWMA, BFWMA), and tissue-sampling method. The tissue-sampling method had two levels: (1) capture occasions that were tissue-sampled with either a bag rinse or a toe clip, or (2) capture occasions that were tissue-sampled with only the swab method. The justification for these two levels was that bag rinses and toe clips were 3.6 times more sensitive than swabs (see results).

We tested all possible combinations of the main effects and the two-way and three-way interaction terms for a total of 2728 models. We used Akaike's information criterion (AIC) to guide model selection. The best model was the one with the fewest parameters and within 1 unit of the lowest AIC score. We used log likelihood ratio tests to determine the statistical significance of the terms in the best model. R (version 2.7.0) was used to analyze the data.

To determine whether temperature influences the prevalence of *Bd*, we obtained mean monthly air temperature data from the Creston Campbell Scientific weather station for the period from 2003 to 2007. The mean monthly air temperature is an average of the mean daily air temperatures for that month. The mean daily air temperature is the average of the maximum and minimum daily air temperature. The Creston Campbell Scientific weather station is less than 20 km from the CVWMA.

#### Survival of *Bd*-infected *R. pipiens*

Mark-recapture analysis estimates the probability of capturing an animal and the probability of survival between capture sessions. Unfortunately there were only 31 recaptures in this study, of which 6 were among years, 3 were among seasons within year, and the remaining 22 were within season (i.e. <24 days). Hence there were not enough recaptures to warrant a proper mark-recapture analysis [31]. Instead, we used a chi-square test to determine whether the return rate (defined as the percentage of captures that are recaptures and which includes both the survival and recapture rate) was different between *Bd*-infected and uninfected *R. pipiens*.

## Results

### Sensitivity of 3 tissue-sampling methods

There were 72 capture occasions where we used more than one tissue sampling method on the same animal. Of these 72 capture occasions, 26 had at least one tissue sample that tested positive for *Bd *(Table 1). Of the 4 *Bd*-positive captures that were both bag rinsed and toe clipped, the bag rinse and toe clip both tested positive in 3 out of 4 captures; in the fourth case only the bag rinse tested positive (Table 1). For lack of more data, we considered the bag rinse and toe clip method to be equally sensitive. There were 25 captures that tested positive for *Bd *according to either the bag rinse or the toe clip method (Table 1). These 25 animals were also swabbed but only 28% of the swabs (7/25) tested positive (Table 1) suggesting that the bag rinse and toe clip methods were 3.6 times more sensitive than the swab method. It was this result that motivated us to include the tissue sampling method in the GLM model of *Bd *prevalence in *R. pipiens *(see Table 2).

**Table 1:** PCR results for *R. pipiens *individuals that were sampled with multiple tissue sampling methods on the same date.

	Tissue samples that test positive for *Bd*								
Method	br/sw/tc	br/sw	br/tc	sw/tc	br	sw	tc	none	Total
BR, SW & TC	1	0	2	0	0	0	0	6	9
BR & SW	***	2	***	***	2	0	***	6	10
BR & TC	***	***	0	***	1	***	0	1	2
SW & TC	***	***	***	4	***	0	14	33	51

Total	1	2	2	4	3	0	14	46	72

The outcomes of the PCR assay for the 72 individuals that were tested with multiple tissue-sampling methods on the same date. The three different tissue sampling methods are bag rinse (BR), swab (SW), and toe clip (TC). The combination of methods used to sample an individual is shown in the far-left column whereas the possible outcomes of the PCR assay are shown in the top-row. For example, of the 9 individuals that were sampled with all three methods (row BR, SW & TC), 1 individual tested positive for all 3 samples (column br/sw/tc), 2 individuals tested positive for both the bag rinse and toe clip (column br/tc), and 6 animals tested negative for all 3 methods (column 'none'). The triple asterisks (***) denote PCR outcomes that are not possible for that combination of tissue sampling methods.

**Table 2:** Generalized linear models (GLM) of the prevalence of *Bd *in *R. pipiens*

#	Model Structure	df	dev	AIC
1	pcr~M + S + Y + T + L + S:T	307	163.19	189.19
2	pcr~M + S + Y + T + L + M:T + M:L + S:T + S:L + T:L + M:T:L + S:T:L	300	149.20	189.20
3	pcr~M + S + Y + T + S:T	308	165.77	189.77
4	pcr~M + S + Y + T + L + M:L + S:T	306	162.15	190.15
5	pcr~M + S + Y + T + L	310	170.36	190.36
6	pcr~M + S + Y + T	311	172.95	190.95
7	pcr~M + S + Y + T + L + M:Y + S:T	305	161.10	191.10
8	pcr~M + S + Y + T + L + M:T + S:T	305	161.11	191.11
9	pcr~M + S + Y + T + L + M:L	309	169.32	191.32
10	pcr~M + S + Y + T + L + M:T + M:L + T:L + M:T:L	304	159.35	191.35

We used GLM with binomial errors to model the prevalence of *Bd *in *R. pipiens *as a function of the tissue-sampling method (M), season (S), year (Y), stage class (T), location (L) and their two-way and three-way interactions. Of the 2728 models tested, we show the 10 models with the lowest Akaike Information Criterion (AIC) scores. Also shown are the residual degrees of freedom (df) and the residual deviance (dev) for each model.

### Prevalence of *Batrachochytrium dendrobatidis *in *Rana pipiens*

**Figure 1:**
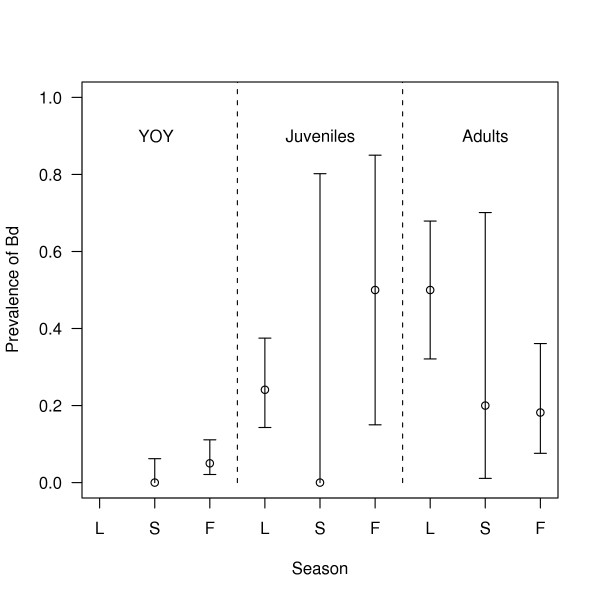
**The prevalence of *Bd *among stage classes and seasons**. The prevalence of *Batrachochytrium dendrobatidis *(*Bd*) in the three stage classes of *Rana pipiens *varies across the three seasons. Shown are the mean prevalence of *Bd *and the 95% confidence limits. The three stage classes are young of the year (YOY), juveniles, and adults and are separated by the vertical dashed lines. The three seasons are spring, summer, and fall and are abbreviated on the x-axis as L, S and F, respectively. Captures are pooled across all years and both locations (CVWMA, BFWMA).

**Figure 2:**
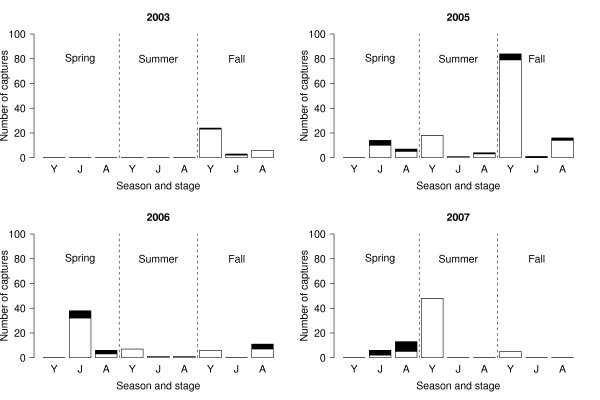
**Infected and uninfected frogs separated by year, stage class, and season**. The number of captures and the prevalence of *Bd *varies among the three stage classes of *R. pipiens *and among the three seasons. The numbers of *Bd*-infected and uninfected captures are shown in black and white, respectively. The three stage classes are young of the year, juveniles, and adults, and are abbreviated on the x-axis as Y, J, and A, respectively. The vertical dashed lines separate the three seasons: spring, summer, and fall. The panels show the four different years: 2003, 2005, 2006, and 2007. Captures are pooled across the two locations (CVWMA, BFWMA).

**Figure 3:**
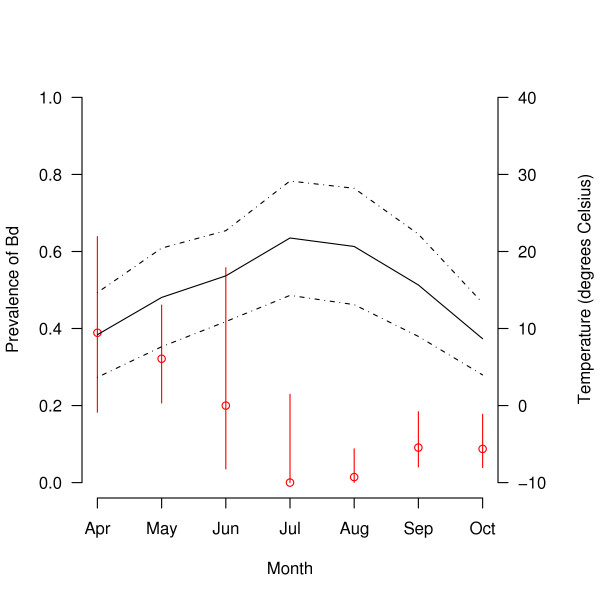
**Prevalence of *Bd *versus the mean monthly temperature**. The prevalence of *Bd *in *R. pipiens *changes across the 7 months of sampling. The red circles show the mean prevalence of *Bd *for each month and the red lines indicate the 95% confidence limits. The solid and dotted black lines show the mean, mean maximum, and mean minimum air temperatures for each month (in °C; averaged for 2003, 2005, 2006 and 2007). Captures are pooled across the three tissue sampling methods (bag rinses, toe clips, and swabs), the three stage classes (young of the year, juveniles, and adults), the four years (2003, 2005, 2006, and 2007), and the two locations (CVWMA, BFWMA).

Over the whole study, there were 320 captures of *R. pipiens*, of which 13.1% (42/320; 95% confidence interval (CI) = 10.0 to 17.8%) tested positive for *Bd*. *Bd *prevalence increased with stage class: young of the year (3.1% = 6/192; 95% CI = 1.3 to 7.0%), juvenile (25.0% = 16/64; 95% CI = 15.4 to 37.7%), and adult (31.3% = 20/64; 95% CI = 20.6 to 44.2%; Figure 1). *Bd *prevalence in the spring (32.1% = 27/84; 95% CI = 22.6 to 43.3%) was much higher than that in the summer (1.3% = 1/80; 95% CI = 0.1 to 7.7%) and fall (9.0% = 14/156; 95% CI = 5.2 to 14.9%; Figure 2). Stage class biased this seasonal pattern as follows. All of the captures in the spring were juveniles and adults whereas most of the captures in the summer and fall were young of the year, which were less likely to be infected with *Bd *(Figure 2). The seasonal pattern of *Bd *prevalence was inversely related with the mean monthly air temperature across the 7 months of sampling (Figure 3). The prevalence of *Bd *in *R. pipiens *was 6.1% (2/33; 95% CI = 1.1 to 21.6%) in 2003, 10.3% (15/145; 95% CI = 6.1 to 16.8%) in 2005, 18.6% (13/70; 95% CI = 10.6 to 30.0%) in 2006, and 16.7% (12/72; 95% CI = 9.3 to 27.7%) in 2007 (Figure 2).

### GLM model of *Bd *prevalence in *R. pipiens*

The best model according to AIC included the tissue sampling method, season, year, stage class and the season:stage class interaction (model 3 in Table 2) but not location. This model provided a good fit to the data (residual df = 308, residual deviance = 165.77). We used log likelihood ratio tests to compare this model to nested models (Table 3A) to determine the statistical significance of each factor (Table 3B) and the parameter estimates (Table 3C). The log likelihood ratio tests found that the tissue-sampling method, season, year, and stage class accounted for a significant portion of the deviance in the prevalence of *Bd *but that the season:stage class interaction was marginally non-significant (Table 3B). The change in deviance from deleting each factor (expressed as a percent of the residual deviance of the best model = 165.77) was as follows: tissue-sampling method (10.9%), season (4.6%), year (5.3%), stage class (6.4%), and season:stage class (4.3%).

**Table 3:** Parameter estimates and statistical significance of the factors that explain the prevalence of *Bd *in *R. pipiens*.

(A) Maximum Likelihood Estimates				
Model	**Model No**.	df	dev	AIC
pcr ~ method + season + year + stage + season:stage	1	308	165.77	189.77
pcr ~ method + season + year + stage	2	311	172.95	190.95
pcr ~ season + year + stage	3	312	191.10	207.10
pcr ~ method + year + stage	4	313	180.54	194.54
pcr ~ method + season + stage	5	314	181.75	193.75
pcr ~ method + season + year	6	313	183.51	197.51

**(B) Likelihood ratio tests**				
**Effect**	**Comparison**	Δ **df**	Δ **dev**	**p**

season:stage	1 vs. 2	3	7.18	0.066
method (swab vs. other methods)	2 vs. 3	1	18.15	<0.001
season (spring, summer, fall)	2 vs. 4	2	7.59	0.022
year (2003, 2005, 2006, 2007)	2 vs. 5	3	8.80	0.032
stage (yoy, juvenile, adult)	2 vs. 6	2	10.56	0.005

**(C) Parameter estimates**				
**Factor**	**Estimate**	**s.e**.	**z**	**p**

Intercept	-4.159	1.241	-3.351	<0.001
method: swab	-2.028	0.488	-4.157	<0.001
season: summer	-1.888	1.125	-1.679	0.093
season: fall	0.549	0.707	0.777	0.437
year: 2005	2.099	0.900	2.333	0.020
year: 2006	2.528	1.015	2.490	0.013
year: 2007	2.704	1.059	2.554	0.011
stage: juvenile	1.705	0.816	2.089	0.037
stage: adult	1.976	0.610	3.239	0.001

We used GLM with binomial errors to model the prevalence of *Bd *in *R. pipiens *(n = 320 captures) as a function of four factors: tissue-sampling method, season, year, and stage class. (A) Shown are the residual degrees of freedom (df), the residual deviance (dev) and the Akaike information criterion (AIC) for six different models. (B) Shown are the likelihood ratio tests between models, the change in the degrees of freedom (Δ df), change in the residual deviance (Δ dev), and the associated p-value (p). (C) Shown are the estimates of the parameters, the standard errors (s.e.), the associated z-scores (z) and p-values (p).

The parameter estimates (Table 3C) confirmed that the prevalence of *Bd *was (1) significantly higher for bag rinses and toe clips than for swab tissue samples, (2) significantly higher in the spring and fall than in the summer, and (3) significantly higher in 2005, 2006, and 2007 than in 2003. After excluding 2003 from the analysis, the factor year was no longer significant, indicating that there were no significant differences in *Bd *prevalence among the last three years of the study.

### Clearance of *Bd *infection in *R. pipiens*

We examined the infection history of recaptured animals to determine whether *R. pipiens *can clear their *Bd *infection. Of the 26 *R. pipiens *individuals that were captured multiple times, 8 frogs tested positive for *Bd *on at least one of their captures. Of these 8 recaptured and *Bd*-infected *R. pipiens*, there were 3 individuals that apparently cleared their *Bd *infection. These individuals tested positive and then negative for *Bd *between consecutive capture occasions (this happened twice in individual RC.06010; Table 4). There were two convincing cases of individuals clearing their *Bb *infection (RC 07003 and RC 06010) where the second negative test was determined by a bag rinse (Table 4). The time interval over which the three individuals cleared their *Bd *infections ranged between 1 and 23 days (Table 4).

**Table 4:** Mark-recapture histories of eight infected *R. pipiens *individuals.

Frog ID	Capture 1	Capture 2	Capture 3	Capture 4
RC.05004	27-Sep-05 (SW)	**11-Oct-05** **(SW)**		
RC.07001	30-May-06 (BR)	**15-May-07** **(BR & SW)**		
RC.05007	28-Sep-05 (SW)	30-Sep-05 (SW)	**18-Oct-05** **(SW)**	
RC.06007	18-Oct-05 (SW)	28-Sep-06 (SW)	**11-Oct-06** **(SW)**	
RC.06008	12-Oct-05 (SW)	4-Jul-06 (SW)	**25-Sep-06** **(SW & TC)**	
RC.06011	**10-May-06** **(SW)**	*11-May-06* *(SW)*		
RC.07003	**15-May-07** **(SW)**	*7-Jun-07* *(BR)*		
RC.06010	**9-May-06** **(BR & SW)**	*30-May-06* *(SW)*	**15-May-07** **(BR)**	*29-May-07* *(BR)*

Shown are the unique frog IDs and the dates of capture as well as the methods used to collect tissue samples for the PCR assay (BR = bag rinse, TC = toe clip, SW = swab). Capture dates and methods where the tissue sample tested positive for *Bd *are in boldface type. Capture dates and methods where the animal recovered from its *Bd*-infection are in italics.

### Survival of *Bd*-infected *R. pipiens*

The return rates (includes both survival and recapture) of *Bd*-infected (9.52% = 4/42; 95% CI = 3.1 to 23.5%) and uninfected *R. pipiens *(9.44% = 27/286; 95% CI = 6.4 to 13.6%) were not significantly different (χ^2 ^= 0.07, df = 1, p = 0.791).

### Ability of surveyors to identify chytridiomycosis in the field

To evaluate the performance of the surveyors we have to consider both type I and type II errors. A type I error occurred when a surveyor classified a healthy frog (as determined by PCR) as infected. A type II error occurred when a surveyor classified an infected frog (as determined by PCR) as healthy. The surveyors had a type I error rate of 9.7% (27/278) and a type II error rate of 71.4% (30/42). These results show that the power to identify *Bd*-infected frogs in the field is low.

## Discussion

The goal of this study was to determine whether *Batrachochytrium dendrobatidis *is threatening British Columbia's endangered population of *Rana pipiens*. From this conservation perspective, the most important result in this study was that the prevalence of *Bd *in *R. pipiens *increased significantly from 2003 to 2005 and then remained stable over the next two years (Table 3C). Surveyors first noticed frogs with chytridriomycosis-like symptoms in 2000 suggesting that the prevalence of *Bd *increased from 2000 to 2005 (Doug Adama, personal observation). Although it is difficult to establish causation, we cautiously suggest that *Bd *caused the 50% decline in the BC population of *R. pipiens *between 2000 and 2005 [24] but that the prevalence of *Bd *has stabilized since 2005.

The results of this study are similar to what happened with the Eungella Torrent frog, *Taudactylus eungellensis*, in Eungella National Park, east-central Queensland, Australia [27]. Between 1985 and 1986, an outbreak of chytridiomycosis is believed to have caused *T. eungellensis *to all but disappear from the park. Six years later, a mark-recapture study found that populations of *T. eungellensis *were persisting with stable, endemic infections of *Bd *[27]. Likewise, in some areas of the Sierra Nevada of California, chytridiomycosis caused the rapid extinction of some local populations of the mountain yellow-legged frog, *Rana muscosa*, while other populations persisted despite high infection levels [32]. These studies suggest that amphibians can evolve resistance to *Bd *and may have the ability to coexist with the disease.

Two other results from this study suggest that *R. pipiens *is coexisting with the disease. Some *R. pipiens *individuals cleared their *Bd *infections (Table 4) and the return rates were not significantly different between *Bd*-infected and uninfected *R. pipiens *suggesting that they had similar survival. Similar results were found in a mark-recapture study on the Stony Creek frog, *Litoria wilcoxii*, where 7 individuals cleared their *Bd *infections and the return rate was not significantly different between *Bd*-infected and uninfected frogs [26]. While the BC population of *R. pipiens *may be coexisting with *Bd*, this does not mean that the population is doing well. Between 2000 and 2005, surveyors have found an average of 10 egg masses per year [24]. Hence *R. pipiens *remains endangered in British Columbia.

The prevalence of *Bd *was higher in juveniles and adults than young of the year, (Table 3C; Figure 1), perhaps because the overwintering ponds are infected with *Bd *and/or sexual transmission between adults in the spring. The results of this study contradict studies on Australian frogs, which found that small frogs were more likely to be infected and carried more intense infections than larger frogs [33,34]. We were surprised that so few young of the year were infected (Figure 1) because they emerge from the same ponds where highly infected adults mate in the spring. One possibility is that the young of the year lose their infections because they emerge from the breeding ponds during the hottest months of the year (July and August; Figure 3). Alternatively, the low proportion of infected young of the year suggests two other possibilities: (1) *R. pipiens *tadpoles are rarely infected with *Bd *or (2) infected *R. pipiens *tadpoles die before they metamorphose. The susceptibility of amphibian larvae to *Bd *has important consequences for the dynamics of amphibian populations [32]. In several amphibian species, surveyors have found dead and dying post-metamorphic individuals in the presence of infected but apparently healthy tadpoles [16,32,35]. In these species, tadpoles may provide an intraspecific reservoir for the disease, which can drive the adult population to extinction [36].

As expected, the prevalence of *Bd *was lower in the summer than in the spring and fall and this was true for all stage classes (Figure 1). This seasonal pattern of lower *Bd *prevalence in the warmer summer than the cooler spring and fall (Figure 3) has been shown in a number of surveys [15,27,37,38]. *Bd *is vulnerable to high temperatures and stops growing at an air temperature of 28°C [7,8]. In addition, in the summer *R. pipiens *adults spend more time foraging in terrestrial habitats whereas in the spring and fall they enter breeding and overwintering habitat where they are more likely to encounter waterborne *Bd *zoospores [23].

This study would have been much improved if we had always used the same tissue-sampling method. We have tried to correct for the tissue-sampling method in our statistical analysis but it is possible that some of our results were biased by the inconsistent tissue-sampling strategy. The bag rinse was equally sensitive at detecting *Bd *as the toe clip method. Together, these two methods were 3.6 times more sensitive at detecting *Bd *than the swab method (Table 1). Surveyors in the present study obtained swabs by rubbing the tip of a sterile cotton swab on the abdomen, thighs, groin, and feet 10 to 20 times [24]. In contrast, Kriger *et al*. swab each frog 70 times and have detected some of the highest prevalences of *Bd *to date [14,26,34,37]. Hence our low swab success rate may be due to the difference in swabbing effort.

## Conclusions

Chytridiomycosis may have caused the recent decline in the BC population of *R. pipiens*. The prevalence of *Bd *appears to have stabilized over the last three years of the study but the population of *R. pipiens *has not recovered. Young of the year emerging from breeding ponds were rarely infected with *Bd *and the prevalence of *Bd *in *R. pipiens *decreased in warmer months. Some individuals cleared their *Bd *infection and the return rate of *Bd*-infected and uninfected individuals was the same suggesting that *R. pipiens *may have evolved resistance to chytrid-related mortality. However, the BC population of *R. pipiens *remains endangered.

## Authors' contributions

MJV analyzed the data and wrote the manuscript. DA and BH designed the survey protocol and conducted most of the fieldwork. JR supervised the PCR assay of the tissue samples. PG provided expertise on amphibians and chytridiomycosis. DA, BH, and PG helped to interpret the data and write the manuscript. All authors read and approved the final manuscript.
